# Effectiveness of mRNA Vaccine Booster against SARS-CoV-2 Infection and COVID-19 in the Adult Population during the First Three Months of the Omicron Wave in Sicily

**DOI:** 10.3390/healthcare11030305

**Published:** 2023-01-19

**Authors:** Giuseppe Vella, Dario Genovese, Miriam Belluzzo, Luca Mazzeo, Vincenzo Pisciotta, Emanuele Amodio

**Affiliations:** Department of Health Promotion, Mother and Child Care, Internal Medicine and Medical Specialties, University of Palermo, Via Del Vespro, 133, 90127 Palermo, Italy

**Keywords:** SARS-CoV-2, vaccine effectiveness, booster dose, Omicron, VOC, public health

## Abstract

Background: In Italy, the administration of the COVID-19 vaccine booster dose started on 27 September 2021, supported by clinical trials corroborating its efficacy. Given the paucity of real-world effectiveness data, this study aims to estimate the vaccine effectiveness of the booster dose against SARS-CoV-2 infection, severe disease, and death in the adult Sicilian population. Methods: This retrospective cohort study was carried out from 1 January to 31 March 2022 and included all residents in Sicily aged ≥ 18 years without previous SARS-CoV-2 infection and with a complete mRNA vaccine primary cycle. The cohort was split into two groups (booster and primary cycle) matched by age, gender, vaccine type, and month of completion of the primary vaccination cycle. Results: 913,382 subjects were observed in the study: 456,690 (50%) were vaccinated with two doses and 456,692 (50%) with three doses. There were 43,299 cases of SARS-CoV-2 among the two-doses vaccinees (9.5%) and 10,262 (2.2%) among the three-doses counterpart. Vaccine effectiveness in the booster cohort was 76.5% and 74.4% against SARS-CoV-2 infection, 85.7% and 79.7% against severe disease, and 84.1% and 73.1% against intubation or death, for BNT162b2 and mRNA-1273, respectively. Conclusions: This study confirmed the remarkable efficacy profile of the SARS-CoV-2 vaccine booster dose against infection, severe disease, and death attributable to the virus. Overall, the results of this study provide important real-world data to support the continued roll-out of the COVID-19 booster dose and have the potential to inform public health policy and guide decisions on vaccination strategies in countries around the world.

## 1. Introduction

The global spread of SARS-CoV-2 has led to an increase in confirmed cases even in countries where a large proportion of the population had been vaccinated with a complete primary cycle [1,2,3]. Several studies have shown a reduction in the effectiveness of primary cycle vaccination against SARS-CoV-2 in terms of infection, hospitalization, and death [4,5,6,7].

Some authors have demonstrated that at least a part of this vaccine effectiveness loss is due to the emergence of new SARS-CoV-2 variants. In particular, the SARS-CoV-2 Omicron variant was able to evade the immunity conferred by COVID-19 vaccines, and more likely to than other variants of concern (VOCs) [8,9,10]. The Omicron strain (B.1.1.529) was first reported to the World Health Organization (WHO) in South Africa on 24 November 2021; since then, it has spread steeply in most countries [11]. In Italy, the proportion of Italian intensive care unit (ICU) beds occupied by COVID-19 patients rose sharply (18.2%) between 3 January and 9 January 2022, with 1622 positive cases detected for 100,000 inhabitants, compared to 9.5% of ICU beds and 195 cases for 100,000 inhabitants between 6 December and 12 December 2021 [12,13]. This upturn coincided with a shift in the prevalent SARS-CoV-2 strain: on 6 December 2021, 99% of the cases were attributed to the Delta variant and just 0.19% to the Omicron variant, which became predominant in less than one month with more than 80% of all cases, until it reached 99% of cases on 31 January 2022 [14,15,16]. According to the previously reported evidence, to re-establish adequate vaccine protection, booster doses were recommended.

Despite noteworthy attempts to control and manage the exponential phase of the pandemic in hospitals with integrated care pathways (ICP), Italy launched a national vaccination campaign on 27 December 2020 [17]. The initial phase of the campaign targeted front-line healthcare workers, who were identified as having a high level of exposure to the virus and a potential role in its transmission. Subsequently, in February 2021, individuals considered to be at high risk of severe COVID-19, such as residents of care homes and their caregivers, and individuals aged 80 years and older, were made eligible for vaccination. Beginning in May 2021, the vaccination was made available to the entire Italian population. After the authorization by the Italian Drug Administration Institute (AIFA) on 27 September 2021, in Italy, the administration of the booster dose of COVID-19 vaccines was introduced, primarily to people older than 80 or residents in nursing homes, regardless of the type of vaccine they had received previously (BNT162b2, mRNA-1273, AZD1222, JNJ-78436735), with one of the two adopted mRNA vaccines (BNT162b2, mRNA-1273) [18]. Progressively, the range of eligible people was enlarged until, on 25 November 2021, the booster dose was offered to all individuals over 18 years old [19]. Eligibility was initially restricted to those who had completed the primary series of two doses of vaccine at least 6 months prior, but this interval was later shortened to 5 months and then to 4 months [20,21,22].

Although several trials have highlighted the strong efficacy of the booster dose in reducing the COVID-19 risk, there are still only a few studies that evaluated the effectiveness of the third dose as real-world data by using a matched retrospective cohort approach. The present study aims to investigate the vaccine effectiveness (VE) of the booster dose of mRNA COVID-19 vaccination by comparing it to the primary series through a retrospective cohort study on all the Sicilian residents over 18 years old in terms of rates of SARS-CoV-2 infection, hospitalization and death.

## 2. Materials and Methods

### 2.1. Data Sources, Study Design and Study Outcomes

We carried out a matched retrospective cohort study on a sample of Sicilian adult individuals, all vaccinated against SARS-CoV-2, to evaluate the vaccine effectiveness (VE) of the booster dose compared to the primary mRNA vaccine cycle in preventing SARS-CoV-2 infection, hospitalization, and intubation/death. 

Sicily is the largest and the fifth most populated region in Italy, with 5 million inhabitants living on the island. In 2020, compared to Italy, its population had both a slight lower average age (44.2 vs. 45.4 years in Italy) and about one percentage point less of people over 65 years old (22.2% vs. 23.3% in Italy). [23,24].

The cohorts under study were followed up for a total period of three months, from 1 January to 31 March 2022.

All the variables were obtained from two different databases, both collected by the Sicilian Regional Health Office under the supervision of the Italian Ministry of Health. Particularly, the first database included information about the Sicilian vaccinated individuals, such as demographic data, the dates of each vaccine shot and the specific vaccine type; the second database provided data on SARS-CoV-2-positive individuals, such as date of infection, sociodemographic and clinical information, hospitalization (eventual occurrence and date), eventual admission to intensive care unit, intubation (eventual occurrence and date), and death (eventual occurrence and date).

According to the Italian National Classification Criteria in terms of COVID-19 severity, we considered as “severe COVID-19” each SARS-CoV-2 positive patient with clinical manifestations of the respiratory tract/other organs that required hospitalization or admission to the intensive care unit [25]. “Intubation/death from COVID-19” status information was obtained from hospital records. For each patient, we considered the worst outcome that occurred as their clinical status.

The two databases were merged, and each participant was linked using the Italian identification code, which is an alphanumeric code of 16 characters that unambiguously identifies individuals irrespective of citizenship or residency status.

The final database allowed us to characterize the individuals for the time of each of the following events: SARS-CoV-2 infection, mild COVID-19, severe COVID-19, and intubation/death. The outcome should be considered as the worst clinical status suffered by each patient during the SARS-CoV-2 positivity period.

We included in the analyses all individuals who met the following criteria:

− Sicilian residency;
− Individuals aged ≥ 18 years; and
− Having completed the COVID-19 primary mRNA vaccine cycle before 31 August 2021.

We excluded from the study individuals that tested positive for SARS-CoV-2 before 1 January 2022.

According to the previously received vaccine/vaccines, each subject was categorized as:

− “Booster dose group”: individuals who received a booster dose from 1 September to 31 December 2021; or
− “Primary cycle group”: individuals who never received a booster dose or received it during the follow-up period (in the latter case, they were considered in the primary cycle group till they were administered the booster dose).

The obtained cohort was split into two matched cohorts in a 1:1 ratio according to sex, year of birth, and month of completion of the primary mRNA vaccination cycle, in order to control for known differences in the risk of exposure to SARS-CoV-2 infection during the follow-up period.

In the time-to-event analysis, for each matched pair, the follow-up started on 1 January 2022 and ended on the date of testing positive for SARS-CoV-2 infection, the date of death, the date when the member of the unexposed group received a booster dose of vaccine (with concurrent censoring of the paired member of the exposed group), or 31 March 2022 (end of the study), whichever came first. The follow-up time was calculated as the number of days from the starting date to the ending date.

Following the aforementioned inclusion/exclusion criteria and the results of the matching, from almost 4 million subjects we obtained a final cohort of 913,382 individuals.

### 2.2. Statistical Analysis

Frequency distributions were used to describe the characteristics of the eligible and matched cohorts. The two cohorts were compared using standardized mean differences, with a standardized mean difference of less than 0.1 indicating adequate matching [26].

Survival plots were constructed with the Kaplan–Meier estimator. The survival (time to event) data for the two groups (booster vs. no booster) were compared using the log-rank test. Hazard ratios (HR) and 95% CIs were calculated by Cox regression analyses comparing rates of SARS-CoV-2 infection, COVID-19 mild cases, COVID-19 severe cases, and COVID-19 death or intubation among those vaccinated with booster dose and those individuals only vaccinated with the primary cycle.

Vaccine effectiveness of the booster dose, compared with that of the two-doses primary series, was estimated with the following equation: Vaccine Effectiveness = (1 − Hazard Ratio) multiplied by 100. All the analyses have been performed with R (version 4.2.2), and a *p*-value < 0.05 was considered statistically significant.

## 3. Results

As reported in Table 1, between 1 January and 31 March 2022 we observed a total of 913,382 individuals aged ≥ 18 years (F:M ratio = 1.2) who received at least a primary m-RNA vaccine cycle against SARS-CoV-2. Among these, 456,690 individuals (50% of the entire cohort) received the booster dose. Most subjects have been vaccinated with BNT162b2 (85.3%) between May and July 2021 (816,643, 89.5%). A total of 80,230 (8.78% of the entire cohort) individuals resulted positive for SARS-CoV-2 PCR-test, most of them in January and March 2022 (63,712, 6.97%), and just 16,518 (1.81%) in February 2022. In the “Primary cycle group”, we observed 43,229 (9.46% of the entire cohort) positive SARS-CoV-2 subjects, 517 (0.11%) severe COVID-19 cases, 9 (0.002%) intubation COVID-19-related cases and 364 (0.08%) COVID-19 deaths. In the “booster dose group”, we observed 38,981 (8.54% of the entire cohort) positive SARS-CoV-2 persons, 201 (0.04%) severe COVID-19 cases, 1 (0.0002%) intubation COVID-19-related case and 127 (0.03%) COVID-19 deaths.

**Figure 1:**
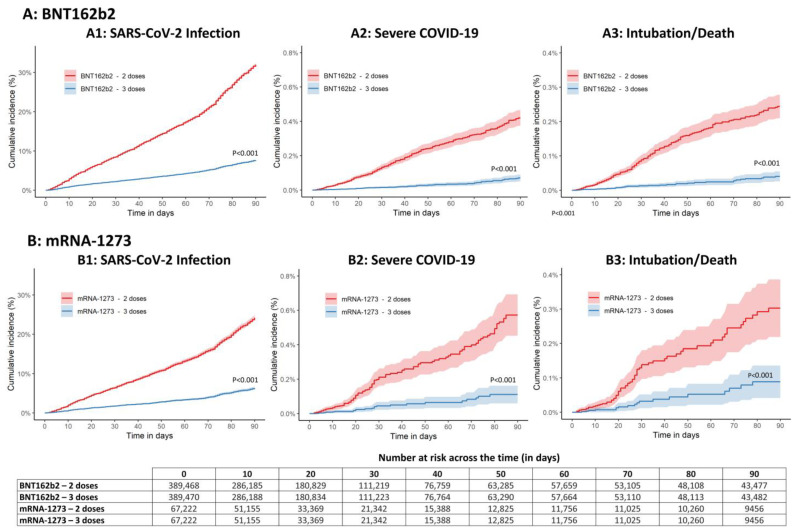
Kaplan–Meier analysis over time (90 days) on SARS-CoV-2 infection (1), severe COVID-19 (2), and intubation/death related to COVID-19 (3) in patients vaccinated with two or three doses of BNT162b2 (**A**) and mRNA-1273 (**B**).

As reported in Figure 1, having received a booster dose with either BNT162b2 or mRNA-1273 showed significant vaccine effectiveness against SARS-CoV-2 infections and against different severity of symptoms including intubation and death (*p* < 0.001).

**Figure 2:**
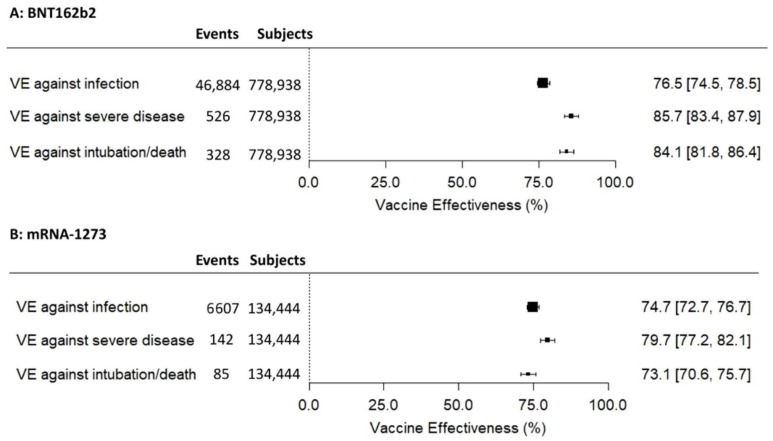
Vaccine effectiveness (VE) of the booster dose against SARS-CoV-2 infection, severe COVID-19, and intubation/death related to COVID-19 in patients vaccinated with BNT162b2 (**A**) and mRNA-1273 (**B**).

In the Cox regression analyses (Figure 2), during the 90 days study period, the BNT162b2 booster dose vaccine effectiveness was of 76.5% against SARS-CoV-2 infection, 85.7% against severe COVID-19 and 84.1% against intubation or death. The mRNA-1273 booster dose vaccine effectiveness was of 74.7% against SARS-CoV-2 infection, 79.7% against severe COVID-19 and 73.1% against intubation or death.

## 4. Discussion

The key findings of the present study strongly suggest that a booster dose of mRNA-based vaccines may reduce the risk of SARS-CoV-2 infection, severe COVID-19, and intubation/death from COVID-19. In fact, the booster dose with mRNA vaccines was associated with at least 74% vaccine effectiveness against infection, and this protection increased when considering more serious outcomes such as severe COVID-19, intubation and death. These results are consistent with those found in other studies. In a study conducted in Qatar, Abu-Raddad et al. [27] observed a booster-dose vaccine effectiveness of 86.1% against symptomatic Omicron infection, which is relatively higher than our results. Similarly, a study carried out in the United Kingdom has reported a vaccine effectiveness of the booster dose of 73.9% against SARS-CoV-2 infection, consistent with our 78% [28]. However, the small variations in results between studies could be attributed to factors such as the prevalence of different COVID-19 variants, prevention strategies and population characteristics (e.g., mask-wearing was mandatory in Italy but not in the UK) [29,30]. In Israel, vaccine effectiveness against severe disease and death from COVID-19 was found to be 92% and 90%, respectively, confirming the soundness of our results against severe outcomes [31].

The importance of the previously reported findings becomes evident if we consider that, according to the previous studies, VE decreases over time after the completion of the main cycle because of the waning of immune protection; thus, the following booster dose could provide enhanced protection [31,32].

Moreover, similarly to the results of the Bar-On et al. [33] study, our vaccine effectiveness against COVID-19 infection was about 74% during the period of Omicron variant dominance. As expected, this is lower compared to the efficacy observed in the clinical trials that were conducted during the circulation of wild-type or Alpha strains. Our results suggest that the booster dose could improve vaccine effectiveness, bringing it closer to that of the primary vaccination cycle against the initial circulating variants. [33,34,35].

Some considerations should also be made in relation to the comparison between the effectiveness of the two investigated mRNA vaccines (mRNA-1273 and BNT162b2). In this sense, it is important to carefully interpret the difference between the two VEs, considering that in Sicily, the mRNA-1273 vaccine was mainly administered to subjects with frail conditions; thus, the relatively lower vaccine effectiveness could be at least in part explained by the higher-risk condition of this group of vaccinees. According to previous considerations, our findings should not be considered or cited as a rebuttal to the current literature, as there is conspicuous research that demonstrated the significantly higher humoral immunogenicity of the mRNA-1273 vaccine compared with the BNT162b2 vaccine, regardless of age category and previous infections. [36,37] This likely happens due to the higher mRNA content in the mRNA-1273 vaccine compared with the BNT162b2 vaccine [38].

Moreover, other limitations should be considered when discussing the results deriving from the present study. First, we did not have PCR analysis confirmation for every case, but we assumed that it followed the trend paved across the Italian country (on 17 January 2022, the Omicron variant for the Italian Institute of Health was 95.8% of the total cases, meanwhile at the end of January 2022 it was 99%) [15,16]. Moreover, since 5 January 2022, a large majority of positive diagnoses have been determined by performing rapid antigen testing, progressively abandoning the usage of PCR technique to diagnose SARS-CoV-2 infection [39]. However, it should be noted that this change towards a less sensitive method of diagnosis did not dramatically affect the investigated cohorts thanks to the broad implementation. For this reason, it cannot be considered a proper limitation of the study.

On the other hand, a strength of this study is the exclusion of individuals with a documented previous infection: the presence of a previous infection could have affected the booster dose protection. However, it should be noted that less than 7% of the population in Sicily at the beginning of the study (January 2022) had a prior infection. As a result, natural immunity to SARS-CoV-2 prior infection may have had a marginal impact on the booster dose vaccine’s effectiveness [40].

Furthermore, we accounted for possible confounding factors such as age and sex in our analysis by matching the cohorts. However, we were unable to account for exposure to risk and behavioral changes following vaccination for the two groups, along with differences in race and ethnicity. The databases used in this study did not contain complete information on the health status of each participant, which prevented us from assessing comorbidities, immunodeficiency disorders or immunosuppressive therapies. Additionally, we were unable to assess lifestyle risk factors such as smoking and alcohol use. Furthermore, we cannot rule out the possibility of bias due to the misclassification of symptom severity, as the data was recorded by different types of healthcare workers with varying levels of clinical expertise. Without data on comorbidities, we could not take into account immunocompromising diseases or drugs, thusly forfeiting a calibration of the strength of vaccine effectiveness with immunocompetence.

Finally, in the rapidly changing scenario observed in Italy during this period, we must put these data into context. Notwithstanding the more than 115 doses administered per 100 inhabitants at the time of the survey, since the new discovery of the Omicron variant and its rapid spread across the whole of Europe, we have observed a precipitous increase in new infections detected since December 2021 [41]; however, afterwards, with a decline in the number of new cases and with a strong sense of protection due to vaccination, it is possible that fewer cases were self-reported in both cohorts.

Studies have investigated the new VOC’s features due to the numerous mutations in terms of contagiousness and related hospitalization, which, in turn, demonstrates its capability of spreading even to fully vaccinated individuals, but with lower rates in terms of hospitalization and deaths [42,43].

Indubitably, vaccine effectiveness evaluation is an important part of understanding the dynamics of immunization against SARS-CoV-2, answering a significant, but not exhaustive, portion of the problem. In the future, a more thorough analysis would benefit from multiparametric serological samples, differentiating N and S1 anti-SARS-CoV-2 antibody production. In fact, it would be helpful to discriminate between the contributions of natural and artificial immunity with respect to low vaccine response or low vaccine effectiveness. Moreover, these data may add crucial information to better study and understand the role of reinfections in eliciting protection.

Based on the findings of our study, it appears that administering a booster dose of the COVID-19 vaccine to individuals who have completed the primary series is an effective means to maintain protection against the virus. In light of the emergence of new variants of SARS-CoV-2, it is crucial to continue monitoring vaccine effectiveness and assessing the need for updated vaccines.

Additionally, our study has implications for the travel industry, international activities, and the overall economic recovery from the pandemic. As countries around the world continue to grapple with the ongoing COVID-19 pandemic, understanding the effectiveness of booster doses could play a crucial role in controlling the spread of the virus and protecting vulnerable populations. Furthermore, our study could also be useful for countries that are still in the early stages of their vaccination campaigns, by providing them with valuable information on the effectiveness of booster doses.

## 5. Conclusions

The assessment of vaccine effectiveness against a new dominant variant, which has a superior intrinsic virulence, is of utmost importance to provide convincing evidence to policymakers in order to drive the booster immunization campaign forward. The results of our study are very clear on the effectiveness of the booster dose, even against the Omicron variant which was not yet known at the time of the mRNA booster administration. The emergence of new variants of concern and the waning of vaccine effectiveness over time should find us already prepared with the best prevention available, which must include full coverage of the population with a booster dose. Overall, the results of this study have the potential to inform public health policy and guide decisions on vaccination strategies in countries around the world.

## Figures and Tables

**Table 1:** Characteristics of the cohort of 913,382 vaccinated subjects aged 18 years or more who received at least a primary mRNA vaccine cycle and were followed up from 1 January to 31 March 2022.

	Population at Risk N (%)	Primary Cycle Group N (%)	Booster Dose Group N (%)
Total	913,382 (100%)	456,692 (100%)	456,690 (100%)
Vaccine type - BNT162b2 - mRNA-1273	778,938 (85.3%)	389,470 (85.3%)	389,468 (85.3%)
	134,444 (14.7%)	67,222 (14.7%)	67,222 (14.7%)
Clinical status - Infection cases - Severe COVID-19 cases - Intubation cases - COVID-19 deaths	913,382 (100%)	43,229 (9.46%)	10,262 (2.24%)
	913,382 (100%)	517 (0.11%)	201 (0.04%)
	913,382 (100%)	9 (0.002%)	1 (0.0002%)
	913,382 (100%)	364 (0.08%)	127 (0.03%)
Gender - M - F	417,449 (45.7%) 495,933 (54.3%)	208,724 (45.70%) 247,966 (54.30%)	208,725 (45.70%) 247,967 (54.30%)
Age - 18 to 30 years - 31 to 40 years - 41 to 50 years - 51 to 60 years - 61 to 70 years - 71 to 80 years - 81 to 90 years - >90 years	94,910 (10.39%) 79,002 (8.86%) 142,248 (15.57%) 197,092 (21.58%) 152,790 (16.73%) 140,216 (15.35%) 90,700 (9.93%) 16,422 (1.80%)	47,455 (10.39%) 39,501 (8.65%) 71,124 (15.57%) 98,546 (21.58%) 76,395 (16.73%) 70,108 (15.35%) 45,350 (9.93%) 8211 (1.80%)	47,455 (10.39%) 39,501 (8.65%) 71,124 (15.57%) 98,546 (21.58%) 76,395 (16.73%) 70,108 (15.35%) 45,350 (9.93%) 8211 (1.80%)
Months of vaccination - April - May - June - July - August	92,206 (10.1%) 278,590 (30.5%) 305,749 (33.47%) 232,304 (25.43%) 4533 (0.50%)	46,104 (10.10%) 139,295 (30.50%) 152,874 (33.47%) 116,151 (25.43%) 2266 (0.50%)	46,102 (10.09%) 139,295 (30.50%) 152,875 (33.47%) 116,153 (25.43%) 2267 (0.50%)
Months of positivity - January - February - March	30,733 (3.36%) 9308 (1.01%) 13,450 (1.47%)	24,252 (5.31%) 7720 (1.69%) 11,257 (2.46%)	6481 (1.42%) 1588 (0.35%) 2193 (0.48%)

## Data Availability

Data will be made available upon request to the corresponding author.
